# The Sternberg Paradigm: Correcting Encoding Latencies in Visual and Auditory Test Designs

**DOI:** 10.3390/vision5020021

**Published:** 2021-05-04

**Authors:** Julian Klabes, Sebastian Babilon, Babak Zandi, Tran Quoc Khanh

**Affiliations:** 1 Laboratory of Lighting Technology, Technical University of Darmstadt, Hochschulstr. 4a, 64289 Darmstadt, Germany; babilon@lichttechnik.tu-darmstadt.de (S.B.); zandi@lichttechnik.tu-darmstadt.de (B.Z.); khanh@lichttechnik.tu-darmstadt.de (T.Q.K.); 2 Light and Health Research Center, Department of Population Health Science and Policy, Icahn School of Medicine at Mount Sinai, One Gustave L. Levy Place, New York, NY 10029, USA

**Keywords:** working memory performance, Sternberg task, memory load, reaction times, differences between visual and auditory encoding

## Abstract

The Sternberg task is a widely used tool for assessing the working memory performance in vision and cognitive science. It is possible to apply a visual or auditory variant of the Sternberg task to query the memory load. However, previous studies have shown that the subjects’ corresponding reaction times differ dependent on the used variant. In this work, we present an experimental approach that is intended to correct the reaction time differences observed between auditory and visual item presentation. We found that the subjects’ reaction time offset is related to the encoding speed of a single probe item. After correcting for these individual encoding latencies, differences in the results of both the auditory and visual Sternberg task become non-significant, p=0.252. Thus, an equal task difficulty can be concluded for both variants of item presentation.

## 1. Introduction

The working memory is an essential metric in visual cognition, information processing, multitasking, and attention deployment [1,2,3,4]. It can be considered as the brain’s ability to temporarily store and recall small amounts of information over brief periods of time and underlies the concept of short-term memory [5]. The amount of information that can be stored before it is lost through decay or interference is limited by the working memory system’s storage capacity [6]. Research on working memory performance has revealed considerable individual differences between humans in terms of information processing and retrieval times [7,8,9,10,11].

To measure working memory performance, a classic paradigm that has been established in cognitive and vision science is the so-called Sternberg task. Originally developed by Sternberg in 1966 [12], it explicitly allows for controlling the memory load filling up the working memory’s storage capacity. In its basic conception, the Sternberg task starts with a memorizing phase during which the memory set, i.e., a string of digits of variable length, is sequentially presented to the subjects, who need to store this information in their working memory. After a delay of a defined number of seconds, which can be considered as the maintenance phase where the information must be kept retrievable in working memory, the actual search phase is initiated. A test digit/stimulus is presented to the subjects who have to decide by a method of forced-choice whether or not this probe was part of the memory set as fast as possible [13].

A distinction can be made between positive trials, where the probe is actually included in the memory set, and negative trials, in which the probe is not included in the memory set [11]. The task difficulty and the subjects’ reaction times (RTs) increase linearly with higher memory load, which is defined by the length of the digit sequence that needs to be memorized. The corresponding rate of increase is the same for both positive and negative trials with only a small offset in RTs between these two conditions [14]: Responses to the latter are in general found to be slower than responses to the former. These findings suggest that information is likely to be retrieved from working memory by some sort of serial exhaustive mental scanning [15] taking place during the search phase, i.e., only after the test stimulus has been compared successively to all memorized items, a response of match or no-match can be given on the basis of a subsequent binary decision process following immediately. Differences in RTs between positive and negative trials can thus be explained by differences in the mean durations of this decision stage for match and no-match results [16].

The Sternberg task has widely been used in the domain of cognitive neuroscience and applied research to infer the neural basis of working memory (e.g., [17,18,19,20,21,22,23,24,25,26]) as well as for the investigation of short-term memory impairment caused by certain pathologies (e.g., [27,28,29,30,31,32]), normal aging (e.g., [33]), or drug use (e.g., [34,35,36,37]). In some of these studies, the Sternberg and Sternberg-like tasks have been adopted as so-called loading tasks to explicitly control the mental workload (or memory load) of the subjects in order to investigate how it affects their performance in a primary task or induces changes on a primary outcome variable.

Depending on the research question that should be addressed, auditory and visual implementations of the Sternberg paradigm have been applied in the literature. For instance, Wickens et al. [38] used an auditory Sternberg task as a diagnostic measure of pilot workload while performing flight maneuvers of different complexity in a flight simulator environment. Okamoto and Nakagawa [39] investigated the effect of exposure to light of different wavelengths on cortical activity responses for a fixed memory load, which was controlled by applying a modified version of the Sternberg memory search paradigm using Japanese four-mora words as auditory stimuli. Tan et al. [40] adopted a visual variation of the Sternberg task using uppercase letters to create memory sets of different lengths and lowercase letters as probe items to examine the impact of an increase in memory load on the arterial blood flow in task-relevant brain areas. In each of these investigations, the Sternberg paradigm served as a tool for manipulating or explaining the outcome variables rather than being the object of study itself.

Lately, the Sternberg paradigm has also been discussed in the context of task-evoked pupil dilation as an index of effort exertion [41]. It has been shown that pupil dilation increases with increasing task demands [42,43,44] and plateaus or even drops when working memory capacity limits are exceeded [45,46]. While some authors have concluded that the observed increase in pupil dilation simply reflects the cognitive load induced by the working memory task, others have argued that the pupil dilation rather represents the cognitive effort recruited in response to these demands [47,48,49]. Studies on inter-individual differences performed by Rondeel et al. [50] and Van Der Meer et al. [51] both suggest that pupil dilation is positively correlated to task performance and, as such, may serve as an indirect measure for the amount of recruited mental effort. Indeed, Bijleveld et al. [52] have shown that subjects exhibit larger pupil dilation when task performance is expected to be more rewarding and difficult.

With regard to such an application framework, in which the pupil response to a simple Sternberg task is suggested to serve as an indirect measure of mobilized mental effort, the task difficulty for a given memory load must be independent of the method of item presentation, i.e., independent of whether the Sternberg task is applied in a visual or an auditory manner. If task difficulty instead depended on the kind of sensory input, inferences on the deviations of mental effort recruitment between different experiments would be strongly limited.

Results of previous studies, however, suggest that the subjects’ reaction times differ dependent on the applied task variant. Chase and Calfee [53], for example, explicitly investigated the effects of auditory versus visual methods of stimuli presentation using memory lists of one, two, or four items randomly generated from three different pre-defined sets of eight acoustically confusable, visually confusable, or neutral roman-alphabetic letters. A further distinction was made between presentation and test mode in such a way that, in addition to the two standard Sternberg variants, visually presented memory lists were combined with acoustically presented probe stimuli and vice versa. Search rates for these combinations were found to be considerably slower compared to using either a pure auditory or pure visual test strategy. In addition, the auditory testing was reported to yield significantly reduced overall reaction times, even though a slightly slower search rate was found for acoustically similar memory lists. Wingfield [54] investigated the effects of the serial position of an acoustically presented probe item in memory lists comprising up to six pre-recorded spoken digits. He reported a significant serial position effect for list lengths of three, four, and six digits, but not for list lengths of two or five. In addition, he compared the results of auditory stimuli presentation to those obtained for two similar visual experiments conducted previously by himself and their co-workers [55,56], where no such serial position effect was observed. RTs in the visual experiments were found to be considerably slower than in the auditory case, even though scanning frequencies were the same.

In a yet unpublished work investigating the impact of different background luminances and light spectra on Sternberg task performance and pupil behavior, we also noticed differences in subjects’ RTs between visual and auditory test designs, but they were opposite to what has been reported in the literature. In addition, we observed differences in the relative pupil size changes as a function of memory load between the two different methods of stimuli presentation. Thus, besides the discrepancies in relation to the literature, it remained unclear whether these findings of task mode dependence reflect a general difference in task difficulty between both test designs or rather a timing artifact due to the involvement of distinct pathways and different kinds of sensory stimulation in the encoding of visual vs. auditory probe stimuli.

**Figure 1:**
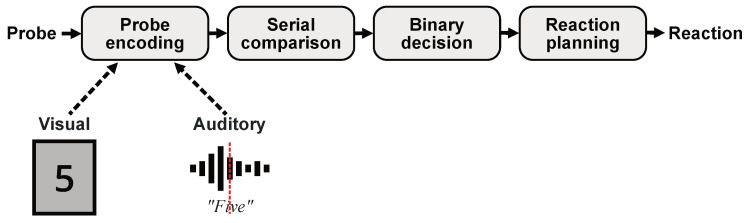
Schematic illustration of Sternberg’s serial exhaustive scanning theory of working memory. It is hypothesized that observed deviations in RTs between visual and auditory test designs can be explained by fundamental differences in the sensory processing of the respective stimuli at the encoding stage rather than by a general difference in task difficulty.

Adopting Sternberg’s serial exhaustive scanning theory, illustrated in Figure 1, the working hypothesis of the present work is that differences between auditory and visual item presentation occur only at the encoding stage without affecting the actual difficulty of the Sternberg task. In the wake of this assumption, visual probe stimuli can be expected to be instantaneously transferred to the memory encoding process at the moment they are perceived by the subject. Auditory probe stimuli, on the contrary, represent continuous signals so that extra time is needed before sufficient sensory information has been accumulated to initiate the mental encoding. Thus, we hypothesize that the observed differences in RTs and pupil behavior can be related to time differences in the mental processing of the different sensory inputs during the encoding phase as additionally indicated in Figure 1. To validate this assumption, we present a new experimental approach that is intended to correct the reaction time differences between auditory and visual item presentation by eliminating the impact of the probe encoding stage. The goal is to show that the general difficulty of a simple Sternberg task is basically independent of the method of item presentation.

## 2. Materials and Methods

### 2.1. Participants

Seventeen paid students (3 female, 14 male) of the Technical University of Darmstadt, Germany, ranging between 19 and 27 years (∅age=23.2, σ=2.7) took part in the experiments. An eighteenth participant that was initially recruited, but attended only one out of two required experimental sessions, and their data were therefore excluded from the analysis. Subjects were instructed to refrain from consuming caffeine and alcohol the evening before and on the day of the experiment. Informed consent was obtained from all participants, and the study was approved by the university’s ethical review committee under reference number EK12/2019. Experiments, data collection, and data storage were conducted in accordance with national and international ethical standards and, in particular, adhere to the Declaration of Helsinki and the requirements of the German Research Foundation (DFG).

### 2.2. Experimental Setup

**Figure 2:**
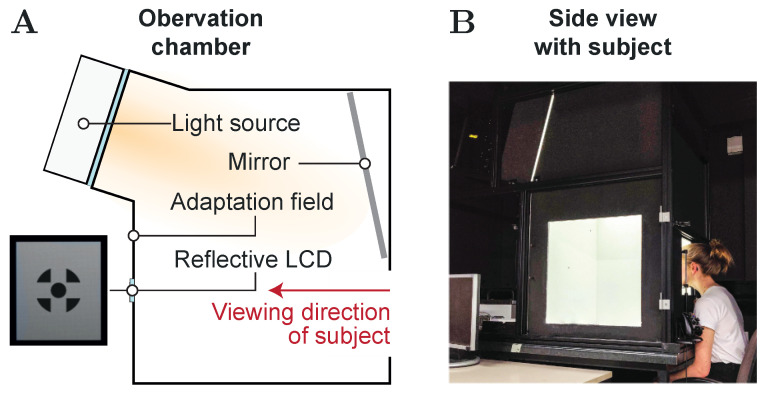
Experimental Setup. (**A**) Schematic illustration of the observation chamber and viewing direction. (**B**) Side view picture of the observation chamber (maintenance door removed) showing a subject focusing the fixation target, which is displayed on the reflective LCD embedded to the center of the chamber’s rear wall. A chin rest is used for positioning and aligning the subject’s head accordingly.

In order to guarantee equal experimental conditions for both the visual and the auditory version of the same simple Sternberg task, an observation chamber setup was used [57,58]. During task performance, as can be seen from Figure 2, the subjects were asked to look into the chamber and keep their eyes focused to a 700 mm × 700 mm adaptation field created by the chamber’s rear wall, while their head was held in position by a chin rest. A small reflective LCD panel embedded to the center of the rear wall at eye level was used for providing a corresponding 0.8° fixation target [59]. Besides ensuring a fixed gaze behavior, the LCD also served for displaying the memory set and probe items during the visual Sternberg task by using single-digit, OpenSans Semi Bold numerals with a height of 20 mm. For the auditory version, on the other hand, the memory set and probe item numerals were presented to the subjects via headphones with active noise canceling in the form of pre-recorded, clearly pronounced audio files. Each of these files was mastered in such a way that it started with the first amplitude of the sound signal and showed a maximum duration of 500 ms.

In order to ensure legibility and an accurate visual processing of the displayed information during the experiments, the adaptation field was homogeneously illuminated with an average illuminance of 500 lx and a correlated color temperature of 4800 K. Temperature-controlled LEDs were used as the light source to ensure stable settings over time. The corresponding task luminance contrast $C_{v}=\frac{L_{B}-L_{T}}{L_{B}}$ was 0.41, where the target (i.e., the displayed item) and background luminance were $L_{T}=13\;\mathrm{cd}\;m^{-2}$ and $L_{B}=22.2\;\mathrm{cd}\;m^{-2}$, respectively. According to Rea’s relative visual performance model [60], accurate processing of visual information can be assumed under these conditions.

For both variants of the Sternberg task, the subjects were instructed to respond to the question of whether or not the probe item was included in the preceding memory set by pushing the right shoulder button of a gaming controller for a match and its left shoulder button for a no-match result as fast and accurate as possible. The controller was connected via USB to the same computer that also controlled the visual and auditory presentation of the memory set and probe items. To accurately measure the subjects’ RTs while taking into account potential latencies of the in- and output devices, a dedicated task implementation in Matlab^®^ based on Brainard et al.’s Psychtoolbox-3 [61,62] was used for proper data acquisition. All experiments were performed and instructions were given in German language.

### 2.3. Test Procedure

For each subject, the test protocol comprised two consecutive experimental sessions. One for visual and the other for auditory stimuli presentation. The sessions were conducted on different days, where the order in which the participants performed the two test versions was randomly counterbalanced. Each session took approximately 30 min and started with the measurement of character-specific response times to obtain a reliable estimate of the corresponding encoding and processing speeds for a later correction of the subjects’ RTs in the actual Sternberg task (see Section 3.2).

For this purpose, a single test item was randomly chosen from L={0;1;2;…;9} and presented to the subjects either visually via the LCD or auditorily over the headphones. In the same way, a randomized sequence of single-digit numerals consisting of five occurrences of the actual test item plus five additional numerals randomly selected from *L* was then presented to the subjects, where an inter-item delay of 2 s was applied. While concentrating on the sequence of numerals, the subjects were asked to press the gaming controller’s right shoulder button as fast as possible every time they recognized the test item within the sequence. RT recording always started with the first amplitude of the item’s respective sound signal or, in the visual case, immediately when it was displayed. The resulting five test item RTs were eventually averaged for each subject, and the whole measurement procedure was repeated for the remaining items in *L*. Subject responses for a given item were considered as lapses and, thus, excluded from further analysis if the corresponding RT values were 2.5 standard deviations (SDs) above or below the item-specific group mean.

**Figure 3:**
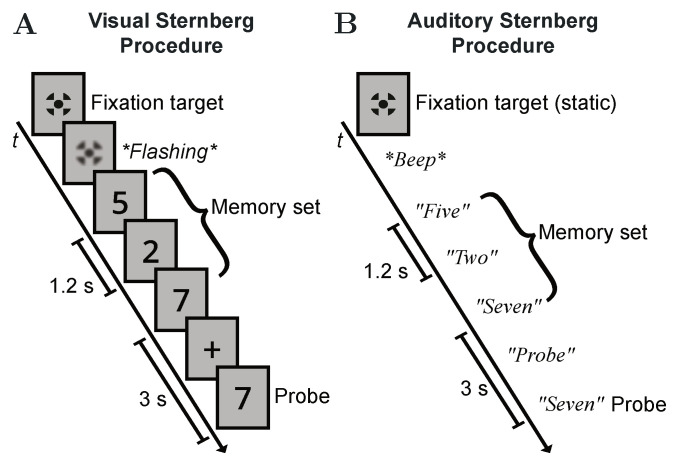
Schematic illustrations of the two Sternberg variants used in this experiment. (**A**) Visual procedure. (**B**) Auditory procedure. In both examples, the memory load is 3 with M={5;2;7} and the positive probe α=7. In the auditory case, the fixation target is displayed continuously.

After measuring the character-specific RTs, a training on the actual Sternberg task, as illustrated in Figure 3, was provided. Each new sequence of a memory set was announced by a short flashing of the fixation target on the LCD display or by a dedicated acoustic signal provided via the headphones. In each of the seven training rounds, a randomly generated memory set *M* of variable length ranging from one to six items with M⊆L was presented to the subjects, one item after the other, for them to memorize the whole sequence. The presentation time of each item (or the inter-item delay in the auditory case) was 1.2 s. After a slightly longer delay of 3 s following the sequence’s last digit, a randomly selected probe item α with α∈L was presented to the subjects, who should indicate whether or not α was included in the memory set by pushing the corresponding button on the gaming controller as fast and accurate as possible. For both variants of the Sternberg task, the appearance of the probe item was indicated during the delay period either by displaying a plus symbol on the LCD display or by a pre-recorded “probe” announcement over the headphones. Again, RT recording was synchronized with the occurrence of the probe item.

Following the accomplishment of all seven training sequences, the actual data acquisition on the Sternberg task was initiated. Here, the general test and randomization procedure was the same as for the training phase with the difference that now, for each level of memory load, the task was repeated six times for both negative and positive trials. Hence, a total number of 72 reaction time measurements (6 memory load levels × 2 trial types × 6 repetitions) were collected per subject. No explicit feedback for correct/incorrect answers was given. Only correct answers were considered for the analysis. Again, the 2.5 SDs criterion was used to define lapses and exclude them accordingly.

### 2.4. Statistical Analysis

Statistical Analysis was performed in R using linear mixed-effects models, where participant was considered as a random factor. For the assessment of character-specific RTs (see Section 3.1), the method of item presentation (visual vs. auditory), as well as the presented numeral itself, were treated as fixed factors. In case of the analysis of the Sternberg data (see Section 3.2), fixed factors were the size of the memory set, i.e., the memory load, the trial type (negative vs. positive trials), the probe item α, and again the method of stimuli presentation (visual vs. auditory). In all cases, the lme() function of the nlme package (version 3.1.147) was used to model the subjects’ RTs based on a maximum likelihood estimation of the regression parameters. The function performs the regression in the formalism introduced by Laird and Ware [63] and allows for nested random effects. Model complexity was increased by adding each factor (and their interactions) as a predictor one at a time to see if the regression significantly improves. Model comparisons of successive complexity stages were performed by calculating the corresponding likelihood ratio, which is asymptotically $\chi^{2}$-distributed. Thus, comparing the observed likelihood ratio to the critical $\chi^{2}$ value for a given significance level serves as an approximate statistical test to indicate whether or not a predictor added to the model has a significant overall effect. Note that a 5% significance level was adopted for all model comparisons performed in this work.

## 3. Results

### 3.1. Character-Specific Encoding Times

**Figure 4:**
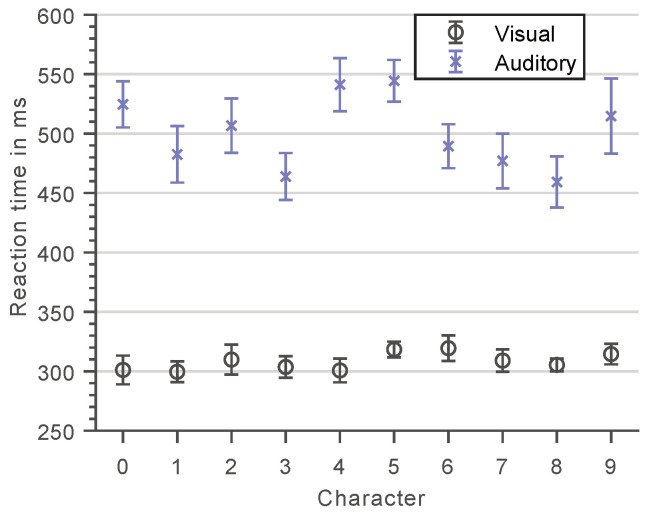
Mean reaction times for the visual (open circles) and auditory (crosses) character specific tests. Error bars represent standard errors.

Figure 4 shows the subjects’ RTs for each of the ten different numerals used as memory set and probe items during the actual Sternberg task for both methods of stimuli presentation. These results reflect the character-specific encoding and processing speeds for recognizing and motorically responding to a single-digit numeral within a sequence of numerals and, thus, approximate the subjects’ base response latencies for a specific probe item presented during the visual or auditory Sternberg task.

As expected, the method of item presentation had a significant effect on the subjects’ base response latencies, $\chi^{2}\left(1\right)=61.7$, p<0.0001, as did the presented numeral itself, $\chi^{2}\left(9\right)=23.6$, p=0.005. While in the visual case all numerals yielded more or less the same mean response latencies, considerably larger deviations are observed for the auditory presentation, $\chi^{2}\left(9\right)=24.3$, p=0.0038, where responses to numerals with similar phonetics of their first consonant (0 & 9 and 4 & 5; in German language) were significantly slower than responses to numerals that are more distinct in their pronunciation, $\chi^{2}\left(1\right)=9.3$, p=0.0023.

On average, subjects respond 192.5 ms faster to visual compared to auditory stimuli, suggesting a more rapid encoding of the former. In addition, smaller variances in RTs are observed between subjects in the visual case. These findings provide evidence that information of a certain complexity (here: numerals) is processed and encoded differently depending on whether it is presented in a visual or auditory manner. This basically indicates the involvement of dedicated pathways and, with regard to the Sternberg paradigm, presumably distinct memory stores. The question that should therefore be addressed as part of the discussion in this work (see Section 4) is whether such fundamental differences in the processing of visual and auditory stimuli also translate to different difficulties of the respective Sternberg variants and, thus, require the recruitment of different amounts of cognitive effort in the anticipation of task fulfillment.

### 3.2. Visual versus Auditory Sternberg Task Performance

**Figure 5:**
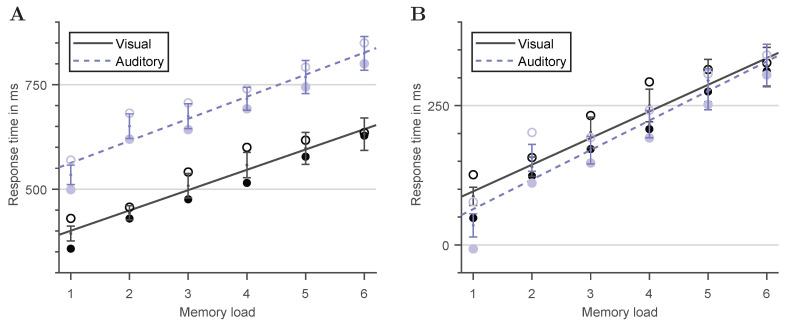
Illustration of the visual and auditory Sternberg task results before and after correcting for character-specific encoding and processing times. Closed circles represent the mean for positive probes, open circles for negative probes. Error bars give the corresponding standard errors. (**A**) Comparison of the uncorrected Sternberg data. Mean RTs rise linearly with increasing memory load, showing equal slopes, but different *y*-intercepts for visual compared to auditory stimuli presentation. (**B**) Comparison of the corrected Sternberg data. Mean RTs still rise linearly with increasing memory load for both methods of stimuli presentation, again showing a similar rate of increase. Differences between the *y*-intercepts become non-significant.

Figure 5A shows the mean RTs for correct match (positive trials) and no-match (negative trials) responses in the Sternberg task for both visual and auditory stimuli presentation as a function of memory load given by the length of the memory sequence. In addition, Table 1 summarizes the mean accuracies for each condition. As can be seen from Figure 5A, the subjects’ RTs in general increase linearly as the number of items to be memorized increases. In all cases, linear regression accounts for more than 93% of the variance of the subjects’ mean response latencies. Furthermore, the rate of increase, which is the inverse of the item scanning frequency, appears to be independent of trial type and the method of stimuli presentation, showing an average value of 50.7 ms per additional item added to the memory set. Differences in RTs are observed only in form of an offset between the different test conditions. Decisions on negative trials took on average 53.2 ms longer than decisions on positive trials, while responses to a visual stimuli presentation were on average 172.8 ms faster than those recorded for the auditory case. Statistical analysis of the RT data revealed a significant main effect of memory load, $\chi^{2}\left(5\right)=96.4$, p<0.0001, a significant main effect of the method of item presentation, $\chi^{2}\left(1\right)=198.9$, p<0.0001, a significant main effect of trial type, i.e., whether or not the probe item was included in the memory set, $\chi^{2}\left(1\right)=42.8$, p<0.0001, and a significant main effect of the probe item, $\chi^{2}\left(1\right)=49.7$, p<0.0001. There were no significant two-way or higher-order interactions, except for a trial type × probe item interaction, $\chi^{2}\left(9\right)=25.1$, p=0.0029, indicating that the differences in reaction times between positive and negative trials differ between probe items.

Linear regression applied to the present RT data set, as illustrated in Figure 5A, shows similar mean slopes of 48.6 ± 8.9 ms per item for the visual and of 52.8 ± 9.2 ms per item for the auditory case, but very distinct *y*-intercepts of 351.9 ± 34.8 ms and 509.9 ± 41.5 ms, respectively. These regression results basically reflect the significant main effect of the method of stimuli presentation and the lack of a significant memory load × method interaction.

## 4. Discussion

The present Sternberg task results mostly agree with those reported in the literature [12,15,16,53,54,55], in particular with regard to the absolute value of the item scanning frequency and its consistency between the different test conditions, with their implications of an equal processing of working memory information during the search phase for both visual and auditory stimuli, as well as a serial exhaustive search strategy underlying, in both cases, the subjects’ final decision process. However, in contrast to the previous findings, the present results suggest in accordance with Section 3.1 a faster mental encoding for visually compared to auditorily presented probe items. This clearly contradicts the results reported by Chase and Calfee [53] and Wingfield [54], who concluded a more rapid processing for the auditory version of the Sternberg paradigm. As an attempt to resolve this discrepancy, it should be stressed that from both of their works, it remains unapparent when and how exactly the RT measurements were triggered in the auditory case. Whereas Wingfield at least stated that in their experiments a voice-operated relay monitoring the auditory output was used to activate the reaction timer, Chase and Calfee provided no information at all regarding their applied RT measurement protocol. Bearing in mind the soft- and hardware limitations of the time when they conducted their experiments, it is very likely that the RT recording started with a certain delay rather than exactly with the first amplitude of the sound signal—as it was ensured in the present case—and, thus, resulted in an underestimation of the subjects’ true Sternberg RTs.

In order to elucidate whether the observed RT offset between visual and auditory stimuli presentation can be explained by a different processing speed during the encoding phase rather than by fundamental variations in task difficulty, the character-specific encoding times discussed in Section 3.1 were used to individually correct each subject’s RT measures. To justify the appropriateness of the proposed correction scheme, we applied Ratcliff’s diffusion model [64,65], which describes the continuous information accumulation during a binary decision process (i.e., whether or not the probe item was included in the memory set) as a random-walk Wiener diffusion process between two decision threshold boundaries assuming a systematic drift component and normally distributed noise [66,67]. In a diffusion model analysis, there are several parameters to be estimated in such a way that the predicted RT distribution matches the empirically measured RT distribution of the subjects as good as possible. In the present case, these parameters are (i) the drift rate ν, which represents the strength and direction of the systematic changes in the diffusion process, (ii) the boundary separation *a*, which reflects the total amount of information considered for a decision defined by the width of the interval between both decision thresholds, (iii) the starting point *z*, which determines an initial bias towards any of the two directions, and (iv) the non-decision time $t_{0}$ and its variability $st_{0}$, which reflect the encoding of the probe stimulus and other decision-unrelated processes. Hence, to verify our assumption that the observed differences in RTs between the two Sternberg variants are likely to be caused by a different processing at the encoding stage and, thus, can be corrected accordingly, it needs to be shown that at each level of memory load the diffusion model parameters ν and *a*, which describe the actual binary decision process, are the same for both visual and auditory stimuli presentation.

The diffusion model parameters were therefore determined for each combination of participant, memory load, and task variant using the fast-dm-30.2 software package [68] provided by the University of Heidelberg. The starting point *z* was fixed to a/2, as there was no reason to expect an initial bias towards any of the two decision threshold boundaries. Table 2 summarizes the estimated mean ± SD diffusion model parameters at each level of memory load for both Sternberg variants. Applying paired *t*-tests with Bonferroni correction at each level of memory load revealed no significant differences between visual and auditory stimuli presentation for the diffusion model parameters ν, *a*, and $st_{0}$. Highly significant differences, on the other hand, were observed for $t_{0}$ (p<0.001 at all levels of memory load). Thus, by applying Ratcliff’s diffusion model, further evidence is given that the observed differences in the RTs obtained for both Sternberg variants mainly occur at the encoding stage, which, as proposed, can therefore be corrected by the individual subject’s corresponding character-specific encoding latencies.

The corrected RT values are eventually plotted in Figure 5B. As can be seen, after this correction, the linear regression lines giving the subjects’ average response latencies for both positive and negative trials virtually fall on top of each other. Differences between the corrected mean slopes for the visual and the auditory case as well as between the corresponding *y*-intercepts become negligibly small. Indeed, statistical analysis re-performed on the corrected data confirms the independence of task mode (i.e., no main effect of the method of item presentation, $\chi^{2}\left(1\right)=1.31$, p=0.252). Thus, an equal task difficulty can be concluded for both Sternberg variants. The deviations observed in the original data in terms of corresponding RT offsets can be attributed to variations of the encoding speed of the probe item, depending on whether it is presented in a visual or an auditory manner.

A potential limitation of the statistical analysis can be identified, due to budgetary constraints, in the relatively small number of participants recruited for the study. Low sample sizes generally reduce statistical power and, therefore, increase the probability of a type-II error, i.e., accepting the null hypothesis when in fact the alternative is true. In the present case, this might limit the confidence in the proposed correction scheme. However, a potentially reduced power in the reported statistical comparisons does not necessarily limit the primary inference of equal task difficulty for auditory versus visual stimuli presentation. In fact, with the uncorrected RT data as a function of memory load being in general accordance with the serial exhaustive mental scanning theory, and with the additional diffusion model analysis revealing no differences in the binary decision process of both Sternberg variants, observed deviations are very likely to be caused by differences during the encoding phase of the probe item, as initially hypothesized. Thus, despite the low sample size, further evidence of the appropriateness of the proposed correction scheme is provided from theory, complying with the results from statistical analysis and the conclusion of the mental effort’s task mode independence.

## 5. Conclusions

In the present work, the Sternberg paradigm was discussed in the context of serving as a probe for measuring the mobilized mental effort with regard to another primary outcome variable (e.g., the pupil diameter). It was argued that in order to ensure generalizability, task difficulty and, therefore, the amount of effort to be recruited for task fulfillment must be independent of the kind of sensory input, i.e., whether the items to be memorized and the probe item are presented in a visual or auditory manner.

However, in accordance with the literature, deviations in the subjects’ RTs between these two methods of stimuli presentation were observed. It was shown that these differences, despite being the opposite direction of what was previously reported, could be explained by generic variations of the encoding speed of the probe item rather than by a change in general task difficulty. Based on the recording of individual, character-specific response and processing times before each subject performed the actual Sternberg task, proper correction of the subjects’ RTs was possible, and the impact of the probe item encoding step could be eliminated. Statistical analysis performed on the corrected Sternberg data thus revealed no significant differences between the working memory processing of visual compared to auditory stimuli presentation so that the hypothesis of equal task difficulty could be confirmed. This work, therefore, provides an essential contribution for any research—vision-related or not—where the Sternberg paradigm is intended to serve as an experimental tool for manipulating or explaining the outcome variables by different means of sensory stimulation.

## Figures and Tables

**Table 1:** Mean accuracies of the subjects’ Sternberg task performance for both visual and auditory stimuli presentation at each level of memory load. Corresponding SDs are given in parentheses.

Memory Load	Visual	Auditory
M1	0.975 (0 .038)	0.995 (0.020)
M2	0.975 (0.048)	0.995 (0.020)
M3	0.975 (0.055)	0.985 (0.043)
M4	0.971 (0.064)	0.990 (0.027)
M5	0.966 (0.050)	0.975 (0.048)
M6	0.912 (0.101)	0.971 (0.040)

**Table 2:** Mean diffusion model parameters ν, *a*, $t_{0}$, and $st_{0}$ for each level of memory load and the two different Sternberg variants. Corresponding SDs are given in parentheses.

	Visual				Auditory			
Memory Load	ν	*a*	$t_{0}$	${st}_{0}$	ν	*a*	$t_{0}$	${st}_{0}$
M1	0.14 (0.48)	0.70 (0.46)	0.29 (0.07)	0.11 (0.07)	0.02 (0.26)	0.67 (0.27)	0.42 (0.08)	0.13 (0.11)
M2	0.33 (0.93)	0.57 (0.32)	0.36 (0.04)	0.16 (0.09)	0.21 (0.67)	0.84 (0.30)	0.47 (0.07)	0.16 (0.11)
M3	0.11 (0.37)	0.67 (0.24)	0.39 (0.07)	0.17 (0.11)	−0.06 (0.26)	0.79 (0.29)	0.52 (0.08)	0.16 (0.14)
M4	0.09 (0.33)	0.74 (0.22)	0.43 (0.09)	0.21 (0.17)	−0.05 (0.17)	0.84 (0.22)	0.55 (0.08)	0.18 (0.12)
M5	0.00 (0.20)	0.73 (0.30)	0.44 (0.08)	0.18 (0.12)	−0.05 (0.11)	0.92 (0.34)	0.56 (0.10)	0.25 (0.09)
M6	−0.03 (0.86)	0.75 (0.42)	0.48 (0.09)	0.17 (0.13)	−0.11 (0.20)	0.97 (0.29)	0.61 (0.11)	0.18 (0.13)

## Data Availability

All data generated or analyzed to support the findings of the present study are included this article. The raw data can be obtained from the authors, upon reasonable request.
